# SARS-CoV-2 Enters Human Leydig Cells and Affects Testosterone Production In Vitro

**DOI:** 10.3390/cells12081198

**Published:** 2023-04-20

**Authors:** Lu Li, Chantal M. Sottas, Hsu-Yu Chen, Yuchang Li, Haoyi Cui, Jason S. Villano, Joseph L. Mankowski, Paula M. Cannon, Vassilios Papadopoulos

**Affiliations:** 1 Department of Pharmacology and Pharmaceutical Sciences, Alfred E. Mann School of Pharmacy and Pharmaceutical Sciences, University of Southern California, Los Angeles, CA 90089, USA; 2 Department of Molecular Microbiology and Immunology, Keck School of Medicine, University of Southern California, Los Angeles, CA 90033, USA; 3 Departments of Molecular and Comparative Pathobiology, Pathology and Neurology, The Johns Hopkins School of Medicine, Baltimore, MD 21205, USA

**Keywords:** SARS-CoV-2, SARS-CoV-2 spike pseudovectors, testis, Leydig cells, testosterone, human, hamster, spike protein, ACE2, TMPRSS2, cinanserin

## Abstract

Severe acute respiratory syndrome coronavirus 2 (SARS-CoV-2), a SARS-like coronavirus, continues to produce mounting infections and fatalities all over the world. Recent data point to SARS-CoV-2 viral infections in the human testis. As low testosterone levels are associated with SARS-CoV-2 viral infections in males and human Leydig cells are the main source of testosterone, we hypothesized that SARS-CoV-2 could infect human Leydig cells and impair their function. We successfully detected SARS-CoV-2 nucleocapsid in testicular Leydig cells of SARS-CoV-2-infected hamsters, providing evidence that Leydig cells can be infected with SARS-CoV-2. We then employed human Leydig-like cells (hLLCs) to show that the SARS-CoV-2 receptor angiotensin-converting enzyme 2 is highly expressed in hLLCs. Using a cell binding assay and a SARS-CoV-2 spike-pseudotyped viral vector (SARS-CoV-2 spike pseudovector), we showed that SARS-CoV-2 could enter hLLCs and increase testosterone production by hLLCs. We further combined the SARS-CoV-2 spike pseudovector system with pseudovector-based inhibition assays to show that SARS-CoV-2 enters hLLCs through pathways distinct from those of monkey kidney Vero E6 cells, a typical model used to study SARS-CoV-2 entry mechanisms. We finally revealed that neuropilin-1 and cathepsin B/L are expressed in hLLCs and human testes, raising the possibility that SARS-CoV-2 may enter hLLCs through these receptors or proteases. In conclusion, our study shows that SARS-CoV-2 can enter hLLCs through a distinct pathway and alter testosterone production.

## 1. Introduction

Severe acute respiratory syndrome coronavirus 2 (SARS-CoV-2), a SARS-like coronavirus [1], continues to produce mounting infections and fatalities worldwide. Beyond causing severe acute respiratory syndrome (COVID-19), SARS-CoV-2 can damage multiple organs, resulting in significant dysfunction. One of the culprits that enables SARS-CoV-2 to affect multiple organs is angiotensin-converting enzyme 2 (ACE2), the main receptor for SARS-CoV-2. Any tissue that highly expresses ACE2, such as the intestinal tract, kidneys, and heart, is significantly susceptible to infection [2]. Recent data point to SARS-CoV-2 infections in the human testis [3,4], which also contain high levels of ACE2. ACE2 expression in the testis is confined to Leydig, Sertoli, peritubular cells and spermatogonia [2,5]. Moreover, low testosterone (T) levels were associated with SARS-CoV-2 viral infections in males [6,7,8,9], suggesting that either systemic low T levels in males may reduce bodily defenses against infections or SARS-CoV-2 viral infections may induce a decline in T levels. Previous studies reported the presence of SARS-CoV-2 dsRNA and nucleocapsid in the testes [10,11]. We detected SARS-CoV-2 nucleocapsid in the interstitial compartment of hamster testes present 4 days after infection, albeit using a different virus titer. With these data in mind, we hypothesized that SARS-CoV-2 could infect human Leydig cells, the main site of T production, and alter their function.

To test our hypothesis, we employed human Leydig-like cells (hLLCs) that were derived from human-induced pluripotent stem cells (hiPSCs) by transiently overexpressing nuclear receptor subfamily 5 group A member 1 (*NR5A1*), which encodes steroidogenic factor 1 (SF-1) and is the master gene for steroidogenesis [12]. The hLLCs express human Leydig cell steroidogenic genes and enzymes essential for T biosynthesis. Their ultrastructure, such as their enriched mitochondria and smooth endoplasmic reticulum, supports their steroidogenic identity. More importantly, hLLCs secreted T in a stimulus-dependent manner, indicating their functional maturation. In addition, hLLCs were derived from hiPSCs, so there are no ethical concerns about human embryonic stem cells.

Herein, we provide evidence that SARS-CoV-2 can enter hLLCs through distinct pathways to affect T biosynthesis. We used a multipronged approach to develop hLLCs as a model for SARS-CoV-2 infection of the testis. We first employed SARS-CoV-2 spike-pseudotyped viral vectors (termed SARS-CoV-2 spike pseudovectors) to show that SARS-CoV-2 can enter hLLCs and affect T production. Further, we tested different SARS-CoV-2 inhibitors to examine the path by which the virus enters hLLCs and showed that SARS-CoV-2 enters hLLCs through pathways distinct from those in Vero E6 cells, a typical model used to study SARS-CoV-2 entry mechanisms. Moreover, we found that NRP1 and CTSB/L were expressed in hLLCs, which may be receptors or proteases specific for SARS-CoV-2. This study demonstrates the complexity of SARS-CoV-2 infection in human health.

## 2. Materials and Methods

### 2.1. hiPSC

The hiPSC line (GM25256*B) was purchased from the Coriell Institute (Camden, NJ, USA). The hiPSC line was established as previously described and passed all tests for human pluripotent stem cells [13]. The hiPSCs were plated on Matrigel-coated six-well plates (Corning, NY, USA) and kept in mTeSR1 medium (Stemcell Technologies, Vancouver, BC, Canada) and 1% P/S (Corning, NY, USA) with daily media changes. The hiPSCs were passed by ReLeSR (Stemcell Technologies, Vancouver, BC, Canada) at ratios of 1:6 to 1:10 every 4–7 days, whenever they reached 80% confluency.

### 2.2. Cell Lines

Adrenal cortical carcinoma H295-R cells were maintained in Dulbecco’s minimum essential medium (DMEM)/F-12 and GlutaMAX with 2.5% NuSerum (Corning, NY, USA) plus 1% P/S. Monkey kidney Vero E6 cells were maintained in DMEM medium supplemented with 4 mM glutamine, 10% heat-inactivated fetal bovine serum (FBS, Sigma-Aldrich, St. Louis, MO, USA), and 1% P/S. Human embryonic kidney 293T cells were maintained in DMEM/F-12, GlutaMAX with 10% FBS, and 1% P/S. All cell lines were passed using 0.25% trypsin/EDTA (Thermo Fisher Scientific, Waltham, MA, USA) at ratios of 1:6 to 1:10, whenever they reached 90% confluency. All cell lines were purchased from ATCC (Manassas, VA, USA).

### 2.3. Plasmids

SF-1-mGFP (#RC207577L2) was purchased from ORIGENE (Rockville, MD, USA). The 18-amino-acid cytoplasmic tail truncated (SΔ18) spike protein from the Wuhan-Hu-1 isolate of SARS-CoV-2 (GenBank accession no. MN908947.3) was provided by James Voss (the Scripps Research Institute, La Jolla, CA, USA) in a pcDNA3.3 plasmid backbone. The G614 mutants were generated by site-directed mutagenesis. A VSV-G protein expression plasmid was obtained from Addgene (Watertown, MA, USA).

### 2.4. hLLC Induction

The hLLC induction was performed as previously described with modifications [12]. The NR5A1/SF-1 gene was cloned from the SF-1-mGFP plasmid and inserted into pLVX-TRE3G-ZsGreen1 (Takara Bio USA, Inc., San Jose, CA, USA) using an In-Fusion HD Cloning Plus Kit (Takara Bio USA, Inc., San Jose, CA, USA). The plasmids contain a Tet-On system, which allows the expression of NR5A1/SF-1 to be controlled by doxycycline (DOX), and fluorescent (ZsGreen1) reporters, which can visualize the expression of NR5A1/SF-1. NR5A1/SF-1-inserted plasmids were used to produce the Tet-On 3G virus using a Lenti-X Packaging Single Shots (VSV-G) kit (Takara Bio USA, Inc., San Jose, CA, USA). The hiPSC lines were co-transfected with the Tet-On 3G virus carrying the NR5A1/SF-1 gene and TRE3G promoter. The hiPSCs inserted with the NR5A1/SF-1-inducible system (NR5A1/SF-1-inserted hiPSCs) were selected by G418 and puromycin. Single colonies were then manually selected. The hiPSCs containing both NR5A1/SF-1 and ZsGreen1 (NR5A1-ZsGreen1) were used for the induction of hLLCs, which were employed in most experiments, including the visualization of SARS-CoV-2’s entry into hLLCs.

The NR5A-1/SF-1-inserted hiPSCs were maintained as general hiPSCs. For hLLC induction, NR5A1/SF-1-inserted hiPSCs were first induced into early mesenchymal progenitors (EMPs) using STEMdiff^TM^-ACF Mesenchymal Induction medium (Stemcell Technologies, Vancouver, BC, Canada) for 4 days and then MesenCult^TM^-ACF Plus medium (Stemcell Technologies, Vancouver, BC, Canada) for 2 days, following the manufacturers’ instructions. On the induction day (ID) 6, EMPs were generated and subsequently cultured in 12-well plates that had been precoated with a solution of human collagen type I. The collagen solution was composed of 50 μg/mL of Human Type I Atelo-Collagen Solution (Advanced Biomatrix, Carlsbad, CA, USA), 10 × PBS, 0.417 mN NaOH, and dH_2_O. Following the addition of 0.3 mL of the collagen solution to each well of the 12-well plates, they were incubated at 37 °C for 1 h. The plates were then washed with PBS three times to remove any unbound collagen. The EMPs were washed with PBS and then seeded onto the coated plates using 1 mL of a gentle cell dissociation reagent. (Stemcell Technologies, Vancouver, BC, Canada) at 37 °C for 8–10 min. The dissociated EMPs were collected into a Falcon tube, centrifuged at 300× *g* for 7 min, and resuspended in hLLC induction basal medium (DMEM/F12, GlutaMAX supplemented with 10% FBS, and 1% P/S) with 10 μM Y-27632 (Stemcell Technologies, Vancouver, BC, Canada). A total of 1 mL of medium containing 1.5 × 10^5^ cells was plated into each well of 12-well coated plates. The cells were cultured at 37 °C for 2 days. From ID 8 to 12, the medium was changed every other day with 1 mL of hLLC induction basal medium with 2000 ng/mL of Dox, 1 mM of N^6^,2′-O-dibutyryladenosine 3′,5′-cyclic monophosphate (dbcAMP, #D0260, Sigma-Aldrich, St. Louis, MO, USA), and 100 ng/mL of desert hedgehog (DHH, R&D Systems, Minneapolis, MN, USA). From ID 14 to 90, the medium was changed every other day with 1 mL of hLLC induction basal medium with DOX, dbcAMP, and DHH, plus 50 ng/mL of human chorionic gonadotropin (hCG, NIDDK, Bethesda, MD, USA). To detect hLLCs with high SF1 expression levels, we performed experiments using hLLCs collected from ID 22, ID 32, and ID82. Our published paper showed that the purity of the terminal hLLC population indicated by the human Leydig cell marker 3*β*-HSD was 93.08 ± 4.21%. We further performed fluorescent-activated cell sorting (FACS) to determine the purity of hLLCs, as indicated by SF-1/ZsGreen1 signals. The FAC results showed that the purity rates of the hLLCs at ID22 and ID32 were 75.16 ± 1.18% and 83.43 ± 1.66%, respectively.

### 2.5. SARS-CoV-2 Spike-Pseudotyped Viral Vector VSVΔG and Bald Vector Production

Vesicular stomatitis virus (VSV) vectors are the most commonly used system for producing SARS-CoV-2 spike-pseudotyped viral vectors (termed here SARS-CoV-2 spike pseudovector) [14]. Replication-deficient VSVΔG vectors [15], deleted for the VSV glycoprotein and containing expression cassettes for firefly luciferase in place of the VSV-G protein, were provided by Jae Jung and Oscar Negrete (Sandia National Laboratories, Albuquerque, NM, USA), respectively. SARS-CoV-2 spike pseudovectors carrying luciferase reporters were used for all experiments.

To generate SARS-CoV-2 spike pseudovectors, 4 × 10^6^ 293T cells were seeded in DMEM plus 10% FBS in a 10 cm poly-lysine-coated plate and transfected with 15 μg of SARS-CoV-2 spike expression plasmid 24 h later, using the calcium phosphate transfection method [15]. The medium was replaced with 10 mL of fresh medium 16 h later; after a further 8 h, 5 mL was removed and replaced with 2 × 10^8^ vector genomes of VSVΔG particles for 1 h at 37 °C. Following incubation, the cells were washed 3 times with PBS and incubated for another 24 h before harvesting.

The vector supernatants were harvested and filtered through 0.45 μm syringe filters and either aliquoted (VSVΔG particles) or concentrated by ultracentrifuge (SARS-CoV-2 spike pseudovector and bald vector) [16]. For each round of ultracentrifugation, the vector supernatants were centrifuged at 17,200× *g* for 90 min at 4 °C. Following centrifugation, the media were carefully decanted into a bleach-filled container. The centrifuge tubes were then loaded with more supernatant for another round of centrifugation under the conditions described above. After 4 rounds of centrifugation, the media were carefully decanted with a pipette to remove the final drop of medium. The centrifuge tubes were then covered in parafilm and stored at 4 °C overnight in an upright position. The following day, the pellets were gently resuspended in 100 μL of PBS by pipetting them 20 times and then stored at −80 °C. The viral titration was performed as previously described [14].

To propagate VSVΔG particles, the same protocol was followed but the SARS-CoV-2 spike expression plasmid was replaced with the same quantity of VSV-G expression plasmid, and no PBS washes were performed after infection by VSVΔG. To generate bald particles, the same protocol was followed but no plasmid was added.

### 2.6. Animals

Male golden Syrian hamster SARS-CoV-2 (SARS-CoV-2 USA-WA1/2020) infections were performed as previously described [17]. At postinfection day (PID) 4, the animals were euthanized with i.p. sodium pentobarbital. A complete postmortem examination with comprehensive tissue harvesting (flash frozen and 10% neutral buffered formalin immersion fixed samples) was performed on all hamsters. The animal procedures in this study were approved by the Johns Hopkins University Institutional Animal Care and Use Committee (approval code: HA20M140; approval date: 5 June 2020) in accordance with the National Research Council’s Guide for the Care and Use of Laboratory Animals (eighth edition) [18]. The Association for Assessment and Accreditation of Laboratory Animal Care International accredits the Johns Hopkins program of animal care and use.

### 2.7. Histology

Hamster testes were immersion-fixed in 10% formalin for 72 h and then kept in PBS at 4 °C until processing. The testes were automatically processed by a Spin Tissue Processor Microm STP-120 (Thermo Fisher Scientific, Waltham, MA, USA) programmed to process tissues (90 min/step) with the following reagents: dehydrant (70%, 80%, 95%, 100%, 100%, 100%) (Richard-Allan Scientific, Canton, MI, USA), 2 cycles of xylene (Cancer Diagnostics, Durham, NC, USA), and 2 rounds of paraffin (Type9, Thermo Fisher Scientific, Waltham, MA, USA). After processing, the testes were embedded using a Tissue Embedding Center EC-350 system (Thermo Fisher Scientific, Waltham, MA, USA) at 60 °C in a tissue block mold filled with hot paraffin. The tissue blocks were cooled down at room temperature (RT) for 30 min and stored at 4 °C until sectioning. The testes were trimmed and rehydrated on ice water for 30 min. After rehydration, 5 µm testis sections were cut using a Rotary Microtome HM-310 system (Thermo Fisher Scientific, Waltham, MA, USA), transferred to a water bath at 37 °C and picked up by VistaVision™ HistonBond^®^ slides (16004406, VWR, Radnor, PA, USA). The sections were dried overnight at RT.

### 2.8. Quantitative Real-Time PCR (qRT-PCR)

The total RNA was prepared using an Ambion^®^ RiboPure^TM^ Kit (Thermo Fisher Scientific, Waltham, MA, USA), and 100 to 500 ng samples were reverse-transcribed into cDNA using PrimerScript^TM^ RT Master Mix (Takara Bio USA, Inc., San Jose, CA, USA). Next, 6–15 ng samples of cDNA were used for the detection of each gene. The qRT-PCR process was performed on a CFX384 Touch^TM^ Real-Time PCR Detection System (Bio-Rad Laboratories, Inc., Hercules, CA, USA), using SYBR^TM^ Select Master Mix (Thermo Fisher Scientific, Waltham, MA, USA) with 100 nM of the forward and reverse primers (Integrated DNA Technologies, Coralville, IA, USA) shown in Table 1. The data were analyzed using Bio-Rad CFX Maestro software. A relative quantification analysis was performed following the 2^−ΔΔCT^ method, and the data were normalized to S18 expression.

### 2.9. Protein Quantification

For the Western immunoblot analysis, the protein concentrations were determined after centrifugation using a Pierce BCA Protein Assay Kit (Thermo Fisher Scientific, Waltham, MA, USA), following the manufacturer’s instructions. For the ELISA analysis and pseudovirus entry assays, the protein concentrations were determined using the Bradford assay as follows. For 96-well plates, 100 mL 5 mg/mL of NaOH was added to each well to lyse the cells, and 20 mL cell lysates were added to a new 96-well plate, followed by 200 mL of Bradford Reagent (Alfa Aesar, Haverhill, MA, USA). The mixture was incubated at RT for 2 min, and the absorbance at 595 nm was measured using a multimode plate reader (Perkin Elmer VICTOR X5, Waltham, MA, USA).

### 2.10. Immunoblotting

The hLLCs, hiPSC, H295-R, or Vero E6 cells were lysed in RIPA buffer with 2% proteinase inhibitor. The human testis lysates were purchased from Novus Biologicals (Centennial, CO, USA). A total of 5 μg of total protein was resolved on 4–20% precast polyacrylamide gels (Bio-Rad Laboratories, Inc., Hercules, CA, USA) and transferred to PVDF membranes (Millipore Sigma, Burlington, MA, USA). After transfer, the membranes were blocked with PBS +0.1% Tween 20 Detergent (PBST) +5% BSA for 45 min and cut into strips to allow the detection of multiple antigens, guided by prestained molecular weight markers (Bio-Rad Laboratories, Inc., Hercules, CA, USA). Membranes were incubated with specific primary antibodies in PBST +5% BSA overnight at 4 °C, washed with PBST 3 times, and incubated with corresponding secondary antibodies for 1 h at RT. The antibodies were then detected using a Clarity Western ECL Substrate system (Bio-Rad Laboratories, Inc., Hercules, CA, USA) and visualized using an Azure c600 Western blot imaging system (Azure Biosystems, Inc., Dublin, CA, USA). Table 2 shows the list of antibodies used.

### 2.11. Fluorescent Immunocytochemistry

Testis slides were deparaffinized using Citrosol for 5 min. For further deparaffinization, rehydration, and unmasking, the slides were placed in Triology solution in a high-pressure cooker for 30–35 min (15 min warm up, 15 min cooking, and 5 min standing), then placed into a second container containing Triology for 5 min. Next, the slides were washed with PBS for 5 min and placed into a beaker containing 95 °C DAKO antigen retrieval solution for 15 min. The container with slides and DAKO antigen retrieval solution was placed at RT for 10 min. The slides were then washed with PBS +0.1% Triton X for 5 min and washed with PBS for 5 min. A DAKO pen was used to make a large circle around each testis section. The slides were then blocked with 10% donkey–goat serum diluted in PBS + 1% BSA for 1 h at RT. Once blocking was done, the blocking buffer was carefully aspirated off. The blocked slides were incubated with anti-SARS-CoV-2 nucleocapsid antibodies diluted in PBS +1% BSA at 4 °C overnight in a humidified chamber. The next day, the slides were washed with PBS for 10 min and incubated with Alexa Fluor™ 647-conjugated secondary antibody (dilution 1:300 in PBS + 1% BSA) for 1 h at RT. After incubation, the slides were washed with PBS twice and mounted using 1 drop of VECTASHIELD Antifade Mounting Medium with DAPI (Vector Laboratories, Inc., Newark, CA, USA). The slides were stored at −20 °C or viewed using a REVOLVE 4 fluorescence microscope (Discover Echo, Inc., San Diego, CA, USA). The exposure times for the DAPI, FITC, and Texas Red channels were set to 100, 70, and 70 ms, respectively.

### 2.12. Cell Binding Assay

The hLLCs were rinsed with PBS twice and fixed with 4% PFA at RT for 30 min. After fixation, the hLLCs were washed with PBS 3 times and blocked with PBS + 1% BSA at 37 °C for 1 h. After washing 4 times with PBS, the hLLCs were incubated with biotin-labeled SARS-CoV-2 S1 protein (Acro Biosystems, Newark, DE, USA) at a concentration of 10 μg/mL diluted in PBS + 1% BSA or blocking solution without S1 protein at 4 °C for 16–24 h. After washing 4 times with PBS, the hLLCs were incubated with fluorescent-conjugated streptavidin diluted in PBS + 1% BSA (1:1000) at 37 °C in the dark for 90 min. The hLLCs were washed with PBS 3 times, stained with DAPI, and viewed using a REVOLVE 4 fluorescence microscope.

### 2.13. SARS-CoV-2 Spike Pseudovector System and Pseudovector-Based Inhibition Assay

In brief, one day before transduction, hLLCs were preseeded on human type 1 collagen-coated 96-well plates at a density of 2.5 × 10^4^ cell/well. Vero E6 and H295-R cells were preseeded at the same density but without the type 1 collagen coating. Before transduction, fresh medium was added. The spike pseudovector, VSVΔG, or bald vector was added to the culture medium and incubated at 37 °C for 1 day. From post-transfection day (PTD) 1 to 5, the supernatants were collected daily for the ELISA analysis and fresh medium was added to the plates.

The transduction efficiency was quantified by measuring the luciferase activity in cell lysates using Britelite Plus (Perkin Elmer, Waltham, MA, USA), following the manufacturer’s protocol. The medium was collected and then refreshed daily for 5 days for the ELISA analysis.

To perform the inhibition assay, the cells were pretreated with SARS-CoV-2 inhibitors, MLN-4760 (ACE2 inhibitor, 16 ng/mL), camostat mesylate (TMPRSS2 inhibitor, 24 mg/mL), and cinanserin hydrochloride (3CL protease inhibitor, 15 mg/mL). The details for the inhibitors are given in Table 3. After 1 h, the pseudovector was added to the medium. The transduction efficiency rates were analyzed the next day.

### 2.14. MTT Cell Proliferation and Cell Viability Assay

The MTT assay was conducted using Cell Proliferation Kit I (Roche, Indianapolis, IN, USA), following the manufacturer’s instructions. Briefly, for a 96-well plate assay, 10 mL of MTT labeling reagent was added to each well and incubated for 4 h at 37 °C. Then, 100 mL of solubilizing solution was subsequently added and incubated overnight at 37 °C. The absorption at 595 nm was measured using a multimode plate reader (Perkin Elmer Victor X5, Waltham, MA, USA).

### 2.15. Fluorescent-Activated Cell Sorting (FACS)

The hLLCs were enzymatically treated with 0.25% trypsin-EDTA for 5 min and collected with DMEM/F12 Glutamax medium with 10% FBS and 1% P/S. The collected hLLCs were centrifuged at 300× *g* for 7 min, and each sample was resuspended with 400 µL of DMEM/F12 Glutamax medium with 3% FBS and 1% P/S. The samples were then filtered into Falcon^®^ 5 mL Round Bottom Polystyrene Test Tubes with Cell Strainer Snap Caps. The samples were analyzed using a BD FACSAria Fusion Cell Sorter and sorted into 1 mL of DMEM/F12 Glutamax medium with 40% FBS and 1% P/S on ice. We set boundaries based on the fluorescent signals of the negative control (NC), which contained EMPs cultured in hLLC induction medium without DOX. Theoretically, the negative cells should not express any ZsGreen1 signal. We set boundaries for the negligible signal (NS) group as 88% of the negative control cells not showing any signal (FITC-A ≈ 1000), and the boundaries for the low-intensity (LI) group were the cells under the threshold (FITC-A ≈ 6000). All cells above this threshold were considered in the high-intensity group (HI). After centrifugation, the sorted cells were plated onto collagen-I-coated plates.

### 2.16. ELISA

The hLLC supernatants were collected. The hormones were quantified using a testosterone ELISA kit (Cayman Chemical, Ann Arbor, MI, USA), according to the manufacturer’s instructions. The quantification of testosterone from samples collected from the SARS-CoV-2 spike pseudovirus entry assay was also done using the same testosterone ELISA kit. The sensitivity of the kit is 6 pg/mL. According to the manufacturer’s instructions, the cross-reactivity rates with different steroids are as follows: testosterone (100%), 19-nortestosterone (140%), 5α-dihydrotestosterone (27.4%), 5β-dihydrotestosterone (18.9%), methyl testosterone (4.7%), androstenedione (3.7%), 11-keto testosterone (2.2%), 5-androstenediol (0.51%), epi-testosterone (0.2%), progesterone (0.14%), testosterone enanthate (0.03%), androsterone (0.05%), androsterone sulfate (0.04%), testosterone sulfate (0.03%), DHEA sulfate (0.02%), estradiol (<0.01%), testosterone glucuronide (<0.01%).

### 2.17. Statistical Analysis

The statistical analysis was performed using GraphPad Prism 9, and statistical significance was determined using a one-way ANOVA followed by Tukey’s multiple comparison test when more than 2 groups were compared. Student’s *t*-test was performed when only 2 groups were compared.

## 3. Results

### 3.1. Interstitial Cells Are the Main Targets of SARS-CoV-2 in the Testis

**Figure 1:**
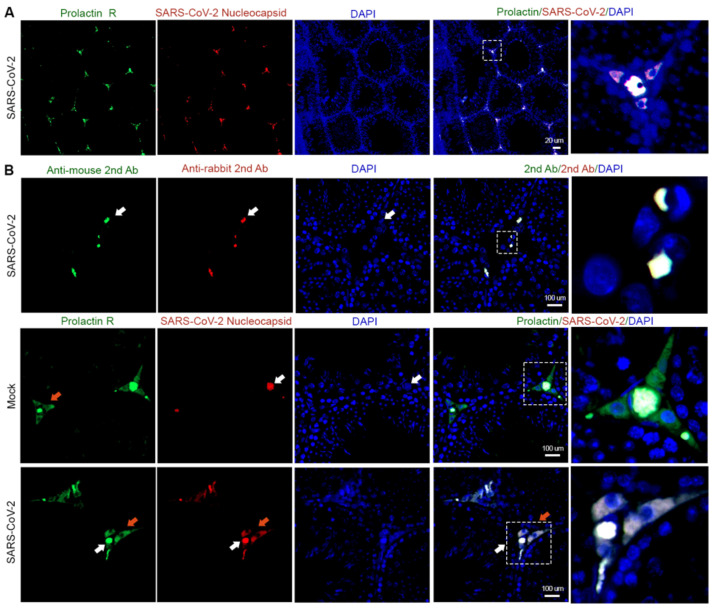
SARS-CoV-2 can infect the Leydig cells from hamster testes. (**A**) At postinfection day (PID) 4, SARS-CoV-2 could be detected by anti-SARS-CoV-2 nucleocapsid antibodies (red signals) in the interstitium. SARS-CoV-2 signals overlap with hamster Leydig cell marker prolactin receptor (Prolactin R, green signals), suggesting that SARS-CoV-2 infected Leydig cells. The area denoted by a white square is enlarged and visualized in the final column of images. (Scale bars: 20 μm) (**B**) Under high magnification, non-specific signals (white arrows) and specific signals from Prolactin R or SARS-CoV-2 nucleocapsid antibodies (orange arrows) can be distinguished. Non-specific signals were caused by second antibodies, or in the mock group overlap with nuclear signals, indicated by DAPI as extremely shining spots. The area denoted by a white square is enlarged and visualized in the final column of images. Non-specific signals in second antibody-stained cells did not completely overlap with cells. In contrast, prolactin receptor and SARS-CoV-2 nucleocapsid antibody signals did overlap with both the Leydig cell cytoplasm and nucleus. Specific signals overlap with the cytoplasm surrounding the nucleus. (Scale bars: 100 μm).

To investigate whether SARS-CoV-2 could infect more than one testicular cell type, we inoculated golden Syrian hamsters with either SARS-CoV-2 USA-WA1/2020 (infected group) or Dulbecco’s modified Eagle’s medium (DMEM, Mock group) via intranasal inoculation. We performed co-immunofluorescent staining with a hamster Leydig cell marker prolactin receptor (Prolactin R) and SARS-CoV-2 nucleocapsid on each hamster’s testicular sections collected at PID 4 [19]. We first confirmed that prolactin receptor antibodies could detect Leydig cells (Figure S1A). We then showed the co-localization of SARS-CoV-2 nucleocapsid signals with Leydig cells in the infected group (Figure 1A). However, we also detected non-specific signals from secondary antibodies in the infected cells (Figure S1B). After further comparison with the mock group, we observed differences between the specific signals and non-specific signals upon enlargement (Figure 1B). The non-specific signals overlapped with the cell nucleus, as indicated by DAPI, as very bright spots (indicated by white arrows). In contrast, the specific signals from prolactin receptor and SARS-CoV-2 were mainly located in the cytoplasm of the cells (indicated by orange arrows), which are oval- or spindle-shaped. These data suggest that SARS-CoV-2 could target Leydig cells residing in the testicular interstitium.

**Figure 2:**
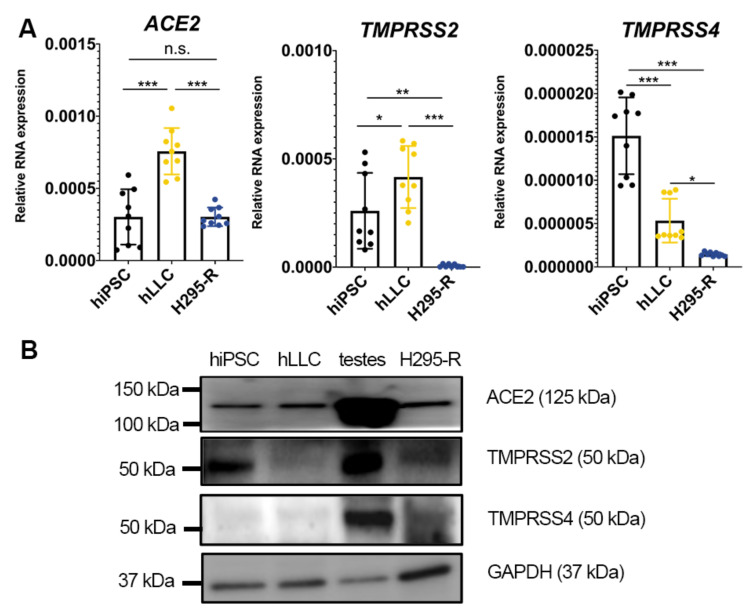
Human testes and human Leydig-like cells (hLLCs) have receptors pivotal for SARS-CoV-2 entry. (**A**) SARS-CoV-2 spike protein receptor *ACE2* mRNA is highly expressed in human-induced pluripotent stem cells (hiPSCs), hiPSC-derived hLLCs, and H295-R human adrenal cortical cells. The SARS-CoV-2 spike protease *TMPRSS2* mRNA is highly expressed in iPSCs, and hLLCs, whereas another SARS-CoV-2 spike protease *TMPRSS4* mRNA is extremely low in hLLCs and H295-R. The hiPSCs used for hLLC derivation were used as controls. Data are presented as means ± SDs, *n* ≥ 3. Not significant = n.s. at *p* > 0.05; * *p* < 0.05; ** *p* < 0.01; *** *p* < 0.001. (**B**) ACE2 protein is expressed in hiPSCs, hLLCs, human testis lysates, and H295-R cells. TMPRSS2 and TMPRSS4 are less expressed in H295-R cells than in human testes. TMPRSS2/TMPRSS4 expression is extremely low in hLLCs, while only TMPRSS4 expression is extremely low in hiPSCs.

As human testis samples are difficult to obtain and Leydig cells from commercial sources neither express the human Leydig cell marker 3β-HSD nor produce T in response to stimulation, we employed hLLCs, the human Leydig-like cell model we previously reported [12], to explore the human Leydig cell tropism of SARS-CoV-2. Due to the overlap in gene and protein expression profiles of steroidogenesis between H295-R cells (human adrenal cortical cells) and Leydig cells [20], we utilized H295-R cells as a suitable reference for examining whether various steroid-producing cells possess similar mechanisms for SARS-CoV-2 entry. ACE2, the receptor for SARS-CoV-2 and a commonly found receptor in multiple tissues, was previously shown to be one of the main culprits enabling SARS-CoV-2 to infect multiple organs. We measured its mRNA and protein levels in hiPSCs, hLLCs, H295-R cells, and human testis lysates. Both the qPCR (Figure 2A) and immunoblotting results (Figure 2B and Figure S2A–C) confirmed its expression in hLLCs, suggesting that the SARS-CoV-2 spike protein could interact with Leydig cells. We then measured the expression of TMPRSS2 and TMPRSS4, the serine proteases found to prime the spike protein and enhance membrane fusion [21]. TMPRSS2/4 was expressed in hLLCs in low-to-no levels compared to human testes and H295-R cells (Figure 2B and Figure S2C,D), implying that SARS-CoV-2 may enter testicular cells and H295-R cells through TMPRSS2/4, while entering hLLCs via an alternative pathway(s).

### 3.2. SARS-CoV-2 Spike Protein Subunit 1 (S1) Can Bind to hLLCs and Alter Their Function

**Figure 3:**
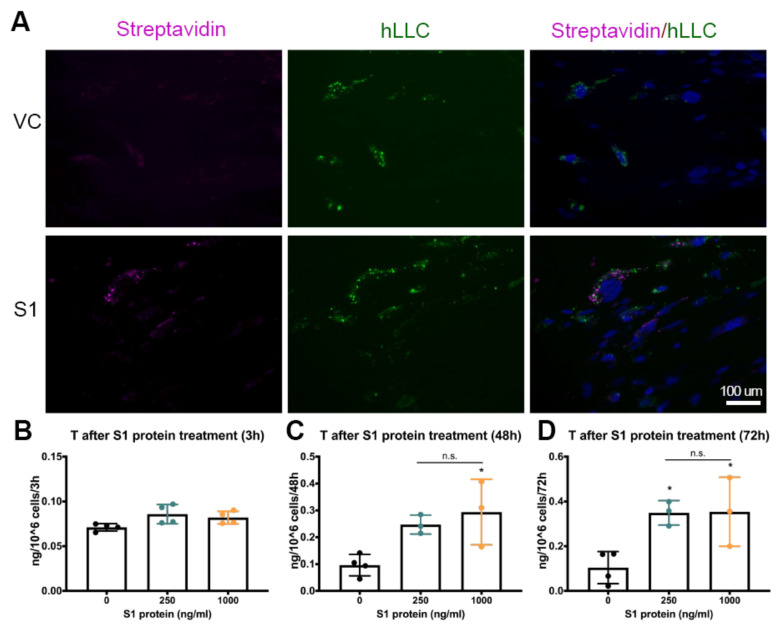
SARS-CoV-2 spike protein subunit 1 (S1) can interfere with T production by hLLCs. (**A**). After fixation, hLLCs were incubated with biotin-labeled SARS-CoV-2 S1 protein (S1 group, 10 μg/mL) or without S1 protein (VC group) for 18 h. Both S1 and VC groups were further incubated with fluorescent-conjugated streptavidin for 90 min. Streptavidin binding to hLLCs is indicated by purple fluorescence. The hLLCs are indicated by green fluorescence inserted during induction. The overlap of purple and green signals suggests that streptavidin can only bind to hLLCs in the presence of S1 protein. (Scale bar: 100 μm) (**B**–**D**) Similar to the experimental conditions in (**A**), hLLCs were treated with 250 or 1000 ng/mL of S1 protein for 3, 48, or 72 h. S1 protein significantly stimulated T production at either 250 or 1000 ng/mL at both 48 h and 72 h. Data are presented as means ± SDs, *n* ≥ 3. Not significant = n.s. at *p* > 0.05; * *p* < 0.05.

Our immunofluorescent staining results showed that SARS-CoV-2 could infect testicular interstitial cells (Figure 1). We, therefore, questioned whether Leydig cells are a direct target of SARS-CoV-2. Spike protein is the major viral antigen that mediates cell entry by binding to ACE2 or other receptors and employing cellular proteases to prime it for membrane fusion [22]. More importantly, it induces a protective immune response in COVID-19 patients. To explore whether SARS-CoV-2 spike protein can bind to hLLCs, we treated hLLCs with biotinylated spike glycoprotein S1, which contains the receptor-binding domain (RBD), followed by fluorescent-conjugated streptavidin treatment to visualize S1 signals. In comparison with hLLCs treated with the medium alone (Figure 3A, upper panel), ZsGreen1-expressing hLLCs (green signals) treated with streptavidin-conjugated S1 protein (purple signals) overlapped or were surrounded by S1 signals (Figure 3A, bottom panel), indicating that S1 protein can bind to hLLCs.

We then assessed whether S1 binding could interrupt hLLC T production. We treated hLLCs with 250 and 1000 ng/mL S1 protein for 3, 48, or 72 h. The short-term treatment (3 h) did not affect T production by hLLCs (Figure 3B). However, the long-term treatments (48 and 72 h) surprisingly and significantly elevated the T production, regardless of the concentration of S1 protein (Figure 3C,D). These results suggest that upon binding, S1 proteins can alter T production by hLLCs.

### 3.3. SARS-CoV-2 Spike Pseudovector Can Enter hLLCs

**Figure 4:**
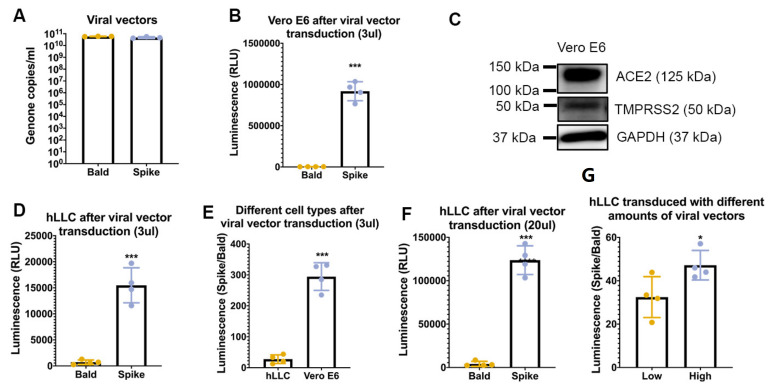
SARS-CoV-2 spike pseudovector can enter ACE2-expressing cells. (**A**) Genome copy number for either SARS-CoV-2 spike protein-containing vector (spike) or vector alone (bald). (**B**) Vero E6 cells were transduced with 3 μL of either bald or spike. Spike pseudovectors entered Vero E6 cells significantly more than bald vectors. (**C**) Vero E6 cells express ACE2 and TMPRSS2. (**D**) The luminescent signals indicate that spike pseudovectors but not bald vectors could enter hLLCs. (**E**) As the entry capacity of bald vectors in hLLCs and Vero E6 cells were different, we used the luminescent signal ratio of spike pseudovectors to bald vectors to evaluate the susceptibility of cells to spike pseudovectors. According to the luminescent signal ratio, hLLCs are less susceptible to spike pseudovectors than Vero E6 cells. (**F**) The hLLCs transduced with 20 μL viral vectors display higher luminescent signal of spike pseudovectors to bald vectors. (G) When the amount of SARS-CoV-2 spike pseudovector was increased from low (3 μL) to high (20 μL), we detected increased ratio of Spike to Bald luciferase signals in hLLCs. This result suggested that hLLCs are more susceptible to high concentrations of spike pseudovectors. Data are presented as means ± SDs, *n* ≥ 3. * *p* < 0.05; *** *p* < 0.001.

After we confirmed the binding of SARS-CoV-2 spike protein to hLLCs, we employed the SARS-CoV-2 spike pseudovector to determine whether SARS-CoV-2 can enter hLLCs. This system was previously used successfully to show the entry capacity of SARS-CoV-2 in HeLa cells expressing ACE2 (HeLa-ACE2) [14]. To confirm that the SARS-CoV-2 spike pseudovector entry capacity was not limited to Hela-ACE2 cells, we tested its transduction efficiency in monkey kidney Vero E6 cells, which are highly susceptible to SARS-CoV-2 [23]. After the SARS-CoV-2 spike pseudovector treatment, the Vero E6 cells were successfully transduced by the luciferase-reporter-expressing SARS-COV-2 spike pseudovector (Figure 4A,B). The immunoblot analysis showed that the Vero E6 cells expressed both ACE2 and TMPRRSS2 (Figure 4C and Figure S2A), confirming the characteristics of SARS-CoV-2 entry into ACE2-expressing cells. Vero E6 cells were employed as a positive control in subsequent SARS-CoV-2 spike pseudovector transduction experiments.

We applied the SARS-CoV-2 spike pseudovector system to hLLCs and successfully detected luciferase signals in hLLCs (Figure 4D). As the bald vectors without the spike protein entered Vero E6 cells more easily than hLLCs (Figure 4B,D, luminescent signals: 3181.0 ± 643.9 vs. 712.0 ± 426.3), we used the luminescent signal ratio of spike to bald vectors to evaluate the entry capacity (Figure 4E). In comparison to Vero E6 cells treated with the same amount of SARS-CoV-2 spike pseudovector, a significantly lower ratio of spike to bald vectors was detected in hLLCs (Figure 4E), suggesting hLLCs are less susceptible to spike pseudovector than Vero E6 cells. When the amount of SARS-CoV-2 spike pseudovector was increased, we detected increased luciferase signals in hLLCs (Figure 4 F,G), implying that the risk of infection in Leydig cells could be elevated with exposure to higher concentrations of SARS-CoV-2 particles.

**Figure 5:**
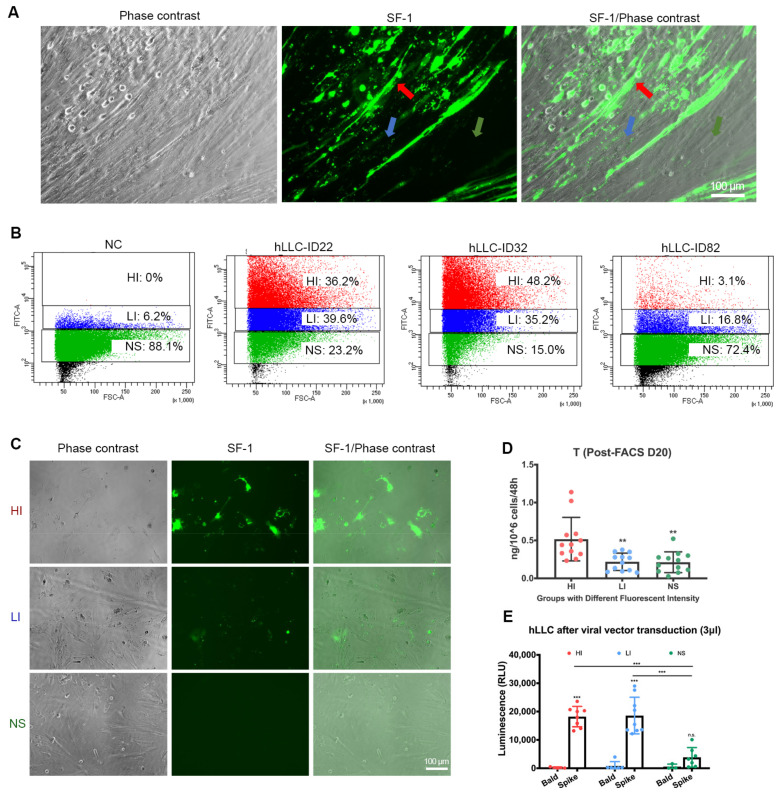
SARS-CoV-2 spike pseudovectors enter hLLCs rather than other induced cells. (**A**) Induced hLLCs are a mixture of cells presenting different ZsGreen1 intensities (green signals). The red, blue, and green arrows point to hLLCs with intense, low or scattered, and negligible ZsGreen1 signals, respectively. (**B**,**C**) The fluorescence-activated cell sorting (FACS) analysis classified mixed hLLCs into high-intensity (HI), low-intensity (LI), and no signal (NS) groups. Early mesenchymal progenitors (EMPs) cultured in hLLC induction medium without DOX were used as the negative control (NC). The hLLCs at induction day (ID) 22, 32, and 82 were used for the FACS analysis. (**C**) After FACS, hLLCs with different ZsGreen1 intensities were cultured in the hLLC induction system and observed under a fluorescent microscope. (**D**) The HI group produced significantly higher levels of T than the LI and NS groups. (**E**) SARS-CoV-2 spike pseudovector can only enter HI and LI groups but not the NS group. Data are presented as means ± SDs, *n* ≥ 3. Not significant = n.s. at *p* > 0.05; ** *p* < 0.01; *** *p* < 0.001.

The NR5A1-ZsGreen1 reporter system enabled us to track hLLCs with varied expression levels of SF-1, which is correlated with steroidogenesis. During induction, we observed that the induced cells presented different levels of NR5A1-ZsGreen1 (Figure 5A). For instance, the cells displayed intense, low or scattered, and negligible signals indicated by red, blue, and green arrows, respectively (Figure 5A and Figure S3). The negligible signal group may have included both hLLCs with low SF-1 expression and non-hLLCs. To investigate whether SARS-CoV-2 spike pseudovectors displayed similarly varied transduction efficiencies in hLLCs with different levels of SF-1 or T production, we performed fluorescent-activated cell sorting (FACS) to select the hLLCs present with varying levels of NR5A1-ZsGreen1 signals, collected from the induction time points ID 22, 32, and 82 (Figure 5B and Figure S4). We used EMPs cultured in hLLC induction medium without DOX as the negative control (NC).

The FACS analyses showed that the induced cells could be divided into three groups based on the intensity of the NR5A1-ZsGreen1 signals (Figure 5B,C and Figures S3B and S4): high expression of ZsGreen1/SF-1 (HI, high-intensity green fluorescent signals), low expression of ZsGreen1/SF-1 (LI, low-intensity scattered green fluorescent signals), and negligible expression of ZsGreen1/SF-1 (NS, negligible signals). We found that the HI group percentage was 83.43 ± 1.66% around ID 32 but decreased by ID 82 (Figure 5B and Figure S4). To confirm that FACS can identify hLLCs with different levels of steroidogenesis, we cultured the sorted groups with hLLC induction medium for 20 more days. We observed that the LI group lost ZsGreen1 signals, whereas the NS group gained a few ZsGreen1 signals, raising the possibility that the LI group represents senescent hLLCs and the NS group represents progenitor hLLCs, which can differentiate into hLLCs under appropriate conditions. This result addressed our concern over the homogeneity of the induction status of the samples we used for other experiments, such as the PCR, Western immunoblotting, and SARS-CoV-2 spike pseudovector entry assay, as we performed all other experiments on samples collected from ID 22 and 32, which had the best green signals. The ELISA analyses of the supernatants showed that the HI group produced significantly more T than the LI and NS groups (Figure 5D), suggesting that the HI group contained mature hLLCs. The intensity of ZsGreen1 only indicates steroidogenic activity in hLLCs, not the purity of the hLLCs. The low-to-no intensity ZsGreen1 cells are still considered hLLCs, as the sorted LI and NS cells can produce a low amount of T (Figure 5D). After the SARS-CoV-2 spike pseudovector treatment, both the HI and LI groups but not the NS group could be transduced with pseudovector (Figure 5E), suggesting that mature hLLCs but not progenitor hLLCs are prone to infection by SARS-CoV-2.

### 3.4. SARS-CoV-2 Spike Pseudovectors Enter hLLCs through a Pathway Different from Those Present in Vero E6 Cells

According to the immunoblotting results (Figure 2B and Figure 4C), TMPRSS2/4 are expressed in Vero E6 cells but at low-to-no levels in hLLCs, raising the question of whether SARS-CoV-2 enters hLLCs through a pathway different from other cells. We, therefore, developed a pseudovector-based inhibition assay by treating Vero E6, hLLCs, and H295-R cells with SARS-CoV-2 spike pseudovectors in the presence of SARS-CoV-2 inhibitors targeting ACE2 (MLN-4760) and TMPRSS2 (camostat mesylate) [24,25]. We also included another inhibitor, cinanserin (hydrochloride), a 3CL protease inhibitor, which was found to have anti-SARS-CoV-2 effects on Vero E6 cells [26].

**Figure 6:**
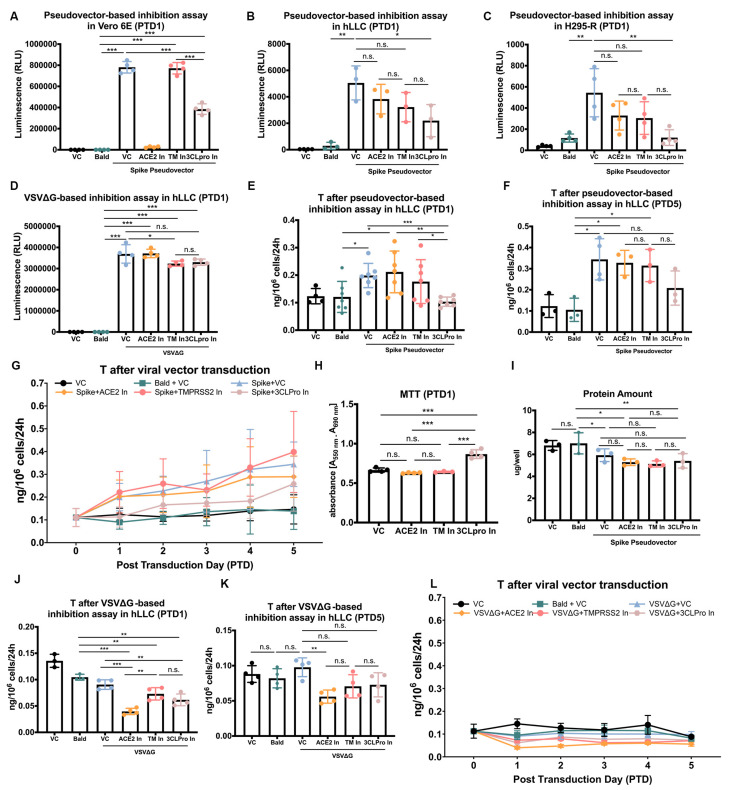
SARS-CoV-2 spike pseudovectors enter hLLCs through pathways different from those in Vero E6 cells. (**A**–**C**) Inhibitors preventing entry of SARS-CoV-2 spike pseudovectors were tested on Vero E6, hLLCs, and H295-R cells. ACE2 inhibitor represses entry drastically in Vero E6 cells, whereas it has only minor effects in hLLCs and H295-R cells. TMPRSS2 inhibitor only slightly affects steroidogenic cells (hLLCs and H295-R) and not Vero E6 cells. The 3CL protease inhibitor (3CLpro In) cinanserin inhibits entry of pseudovector in all cell types. (**D**) Inhibitors were tested against entry of VSVΔG vector in hLLCs. ACE2 inhibitor (ACE2 In) shows no inhibitory effect. TMPRSS2 inhibitor (TMPRSS2 In) significantly inhibits VSVΔG entry, while 3Clpro In slightly inhibits VSVΔG entry with no significance. (**E**–**G**) From PTD 1 to 5, spike pseudovector entry into hLLCs significantly promoted T production. In the presence of the three inhibitors with spike pseudovector transduction, only 3Clpro In could repress the stimulatory effects of SARS-CoV-2 spike pseudovector entry on T production. (**H**) The MTT assay results show no statistically significant differences between VC and ACE2 In or TMPRSS2 In, suggesting these inhibitors had no effect on hLLC growth or viability. However, the hLLC viability is significantly higher under 3CLpro In treatment compared to VC, ACE2 In, or TMPRSS2 In. The lack of 3CL protease expression in hLLCs implies that 3CLpro In (cinanserin) may play a role in promoting hLLC viability or stimulating hLLC growth, even though the contribution of 3CL protease to these processes may be negligible. (**I**) At PTD 5, SARS-CoV-2 spike pseudovector entry represses cell growth with or without inhibitors. (**J**–**L**) Comparing PTD 1 to 5, VSVΔG entry of hLLCs show no significant effect on T production by hLLCs. In the presence of the three inhibitors with VSVΔG transduction, T production by hLLCs is significantly inhibited at PTD 1 in comparison to the bald group. Data are presented as means ± SDs, *n* ≥ 3. Not significant = n.s.; *p* > 0.05; * *p* < 0.05; ** *p* < 0.01; *** *p* < 0.001.

The bald vector (bald)-treated Vero E6, hLLCs, and H295-R cells showed similar levels of luminescent signals to cells cultured under normal conditions (VC, vehicle control) (Figure 6A–C). The luminescent signals were significantly higher in all three cell types treated with SARS-CoV-2 spike pseudovectors compared to VC (Figure 6A–C). The ACE2 inhibitor (ACE2 In) substantially blocked the spike pseudovector entry into Vero E6 cells (96.7 ± 0.7%) (Figure 6A), whereas it had only mild inhibitory effects on hLLCs (24.7 ± 8.2%) and H295-R cells (39.8 ± 8.9%) (Figure 6B,C). The TMPRSS2 inhibitor (TM In) showed no impact on Vero E6 cells (1.2 ± 2.6%) (Figure 6A), indicating that SARS-CoV-2 may enter Vero E6 cells through other proteases. However, the TMPRSS2 inhibitor had moderate effects on hLLCs (37.2 ± 6.3%) and H295-R cells (45.6 ± 10.1%) (Figure 6B,C), implying that the priming effects of TMPRSS2 on the spike protein might be limited by its low expression in these cells (Figure 2A,B). Cinanserin, an inhibitor of 3CL protease inhibitor (3CLpro In), the main protease of SARS-CoV-2, inhibited the entry of spike pseudovector in all three cell types, although not equally (hLLC: 45.5 ± 19.9%; H295-R: 75.8 ± 12.3%; Vero E6: 50.8 ± 3.2%) (Figure 6A–C). These results suggest that SARS-CoV-2 may enter hLLCs through a pathway similar to H295-R but not Vero E6 cells.

Since cinanserin was shown to inhibit the 3CL protease expressed by SARS-CoV-2 viruses [26] but the SARS-CoV-2 genome is not included in SARS-CoV-2 spike pseudovectors, the question becomes whether cinanserin (3CLpro In) targeted the VSVΔG genome or the spike protein itself. We compared the entry efficiency of VSVΔG in hLLCs treated with or without cinanserin, ACE2 inhibitor, or TMPRSS2 inhibitor (Figure 6D). The comparison showed that the ACE2 inhibitor showed no effects on VSVΔG entry (−1.3 ± 7.3%); TMPRSS2 inhibitor showed slight but significant inhibitory effects (11.7 ± 7.5%), and the 3CL protein inhibitor showed slight but not significant inhibitory effects (11.1 ± 2.1%) (Figure 6D). As TMPRSS2 was expressed in low-to-no levels in hLLCs and TMPRSS2 is a cellular type II transmembrane serine protease (TTSP), it is possible that the TMPRSS2 inhibitor camostat mesylate can target other TTSPs in hLLCs [27].

### 3.5. SARS-CoV-2 Spike Pseudovector Entry Dysregulates T Biosynthesis of hLLCs

The binding of S1 protein to hLLCs was shown to enhance T production in hLLCs, indicating that the entry of SARS-CoV-2 may lead to alterations in Leydig cell function. We evaluated the T production following the SARS-CoV-2 spike pseudovector treatment. Similar to the effects of S1 protein on hLLC function, the transduction of SARS-CoV-2 spike pseudovectors significantly increased the T production in hLLCs at post-transduction day 1 (PTD 1) (Figure 6E). ACE2 and TMPRSS2 inhibitors, which could not significantly block the pseudovector entry (Figure 6B), did not block the increased T production. In contrast, the 3CL protease inhibitor reduced the T levels drastically to closer to those of hLLCs treated with bald vectors. This result suggests that the 3CL protease inhibitor may have blocked SARS-CoV-2 spike pseudovector entry enough so that the concentration of pseudovectors in hLLCs was insufficient to enhance T biosynthesis.

To track the later effects of SARS-CoV-2 spike pseudovector entry on hLLCs, we analyzed T production in transduced hLLCs from PTD 0 to 5. To reveal the relationship between spike pseudovector presence and T levels in hLLCs, we evaluated the luciferase signals from spike pseudovectors and T production simultaneously, collecting the medium and adding fresh medium every day after vector transduction. The hLLCs treated with VC or bald vectors showed steady T levels for 5 days (Figure 6E–G). However, the T levels from hLLCs transduced with SARS-CoV-2 spike pseudovector alone or co-treated with ACE2 or TMPRSS2 inhibitor increased from PTD 0 to 5 (Figure 6E–G), indicating a stimulatory effect for SARS-CoV-2 spike pseudovector entry. Pseudovector-transduced hLLCs treated with 3CL protease inhibitor showed small increases in T levels at later times (Figure 6G) when such levels became similar to those of hLLCs treated with ACE2 inhibitor (Figure 6F), suggesting that the presence of pseudovectors in hLLCs, even in low amounts, can affect normal T biosynthesis if they exist for a sufficient time.

To exclude the possibility that the increases in T levels were not due to proliferation stimulation or cell viability enhancement by SARS-CoV-2 inhibitors, we performed an MTT assay. The MTT assay showed that only the 3CL protease inhibitor stimulated hLLC proliferation or increased hLLC viability (Figure 6H). Given the absence of 3CL protease expression in hLLCs observed in our results, it is possible that the 3CL protease inhibitor (cinanserin) plays a role in promoting hLLC viability or stimulating hLLC growth, while 3CL protease does not contribute to these processes. In addition, we performed a Bradford protein assay at PTD 5 to evaluate the total protein in hLLCs under each treatment condition. In comparison with the bald-vector-treated group, the SARS-CoV-2 spike pseudovector-transduced groups showed less protein, suggesting that pseudovector entry might inhibit hLLC growth or have cytotoxic effects (Figure 6I). Moreover, the protein amounts were similar between pseudovector-transduced groups treated with different inhibitors, indicating that individual inhibitors did not counteract the inhibitory effects of the pseudovector on hLLC growth (Figure 6H,I). It is possible that the presence of the VSVΔG genome can stimulate T production by hLLCs. We, therefore, tracked the T production by hLLCs following the treatments with bald and VSVΔG vectors with or without the three inhibitors (Figure 6J–L). In contrast to the stimulatory effects of spike pseudovectors, VSVΔG vector transduction showed no effects on T production compared to the bald group (Figure 6J–L). The co-treatment of VSVΔG with inhibitors showed inhibitory effects on T production compared to the bald group at PTD 1 (Figure 6J). These results imply that the stimulation of T production is caused mainly by the presence of SARS-CoV-2 spike pseudovectors rather than the VSVΔG genome.

Taken together, these results suggest that SARS-CoV-2 spike pseudovector entry can stimulate T production in hLLCs. Furthermore, these results imply that SARS-CoV-2 infection of the testis has the potential to dysregulate T biosynthesis.

### 3.6. Human Testis and Leydig-like Cells Present Other Receptors or Proteases for SARS-CoV-2 Entry

The entry mechanisms of SARS-CoV-2 have been extensively studied in other cell types, providing a comprehensive theoretical basis to understand SARS-CoV-2 infection in the human testis. However, differences between cell types, such as the differential expression of spike protein receptors or proteases and their binding affinities to spike protein, may lead to conflicting results. The low-to-no expression levels of TMPRSS2 and TMPRSS4 in hLLCs suggest that hLLCs may possess another cell-specific SARS-CoV-2 tropism.

**Figure 7:**
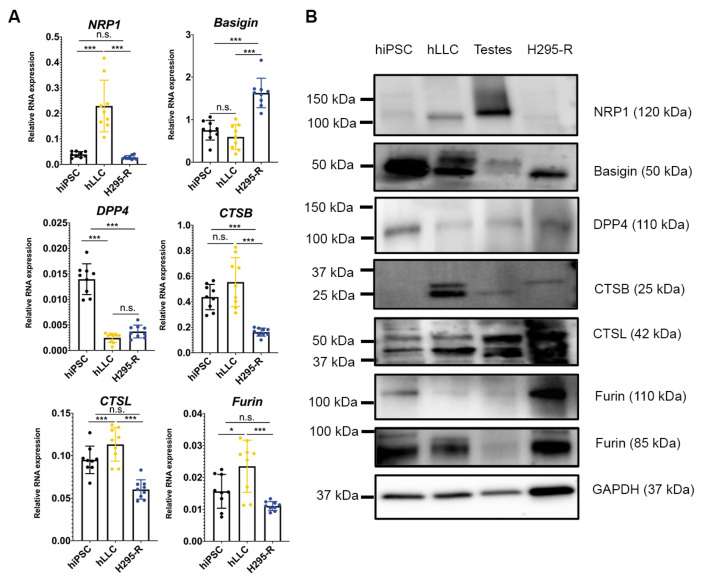
Potential pathways of SARS-CoV-2 entry in hLLCs. (**A**) The qPCR analyses show that SARS-CoV-2 receptors, including neuropilin-1 (NRP1), basigin, and dipeptidyl peptidase-4 (DPP4), and potential SARS-CoV-2 proteases, including cathepsin B (CTSB), cathepsin L (CTSL), and furin, which are responsible for spike cleavage, are highly expressed in hLLCs and H295-R cells. The hiPSCs used for hLLC derivation were used as a control. Data are presented as means ± SDs, *n* ≥ 3. Not significant = n.s. at *p* > 0.05; * *p* < 0.05; *** *p* < 0.001. (**B**) NRP1, basigin, DPP4, CTSB/L, and furin are all expressed in hLLCs and human testes, whereas NRP1 is not present in H295-R cells and hiPSCs. Moreover, CTSB is not expressed in hiPSCs, suggesting that entry pathways of SARS-CoV-2 do not fully overlap between hLLCs, H295-R cells, and hiPSCs.

To identify proteins with the potential to interact with and prime the SARS-CoV-2 spike protein, we assessed the expression of other reported SARS-CoV-2 receptors, neuropilin-1 (NRP1) [28], basigin [29], and dipeptidyl peptidase-4 (DPP4) [30], as well as SARS-CoV-2 proteases, cathepsin B (CTSB) [31], cathepsin L (CTSL) [32], and furin [33]. The qPCR results showed that all of them were expressed in steroidogenic hLLCs and H295-R cells (Figure 7A). Consistent with the mRNA expression in these cell types, NRP1, CTSB, and CTSL were expressed at higher levels in hLLCs compared to H295-R, while DPP4 and furin (85-kDa band) were similarly expressed in both cell types, as observed from their mRNA expression levels. However, basigin showed a differential protein expression pattern; the mRNA of basigin had higher expression levels in H295-R cells, whereas its protein expression was higher in hLLCs. Additionally, although furin’s mRNA expression was higher in hLLCs, 110 kDa furin was expressed at lower levels in hLLCs compared to H295-R cells. This discrepancy between the mRNA and protein expression levels has been reported previously and is likely due to differential rates of production and degradation between mRNA and proteins [34]. All receptors and proteases were present in human testes, but NPR1 and CTSB were not present in hiPSCs (Figure 7B).

Taken together, these results suggest that the human testis and Leydig cells may present pathways for SARS-CoV-2 entry that are different from other steroidogenic cell types and other SARS-CoV-2-targeted organs.

## 4. Discussion

SARS-CoV-2 infection of human testicular tissue and abnormal postinfection levels of sex hormones have been observed [4,6]. Low T levels were found to be an indicator of the most severe clinical outcomes for male COVID-19 patients [6]. However, whether low T was present prior to infection or the result of infection has not been studied. Several groups have shown SARS-CoV-2 virions in the testes [4,10,11,35]. However, there is limited evidence for SARS-CoV-2 infection of human Leydig cells, which produce T in males [36] and which may explain the alteration of sex hormones in some COVID-19 patients. Herein, we detected SARS-CoV-2 in the interstitial compartment of golden Syrian hamster testes, consistent with data from two other studies [10,11]. Moreover, we showed that SARS-CoV-2 nucleocapsid signals co-localized with Leydig cells, suggesting they specifically target Leydig cells in the testis. As testicular tissue from SARS-CoV-2-infected men is hard to obtain and inadequate for the study of pathogenic mechanisms, we employed a human Leydig-like cell model, previously established by our laboratory, to study SARS-CoV-2 entry mechanisms [12]. Our FACS analysis in this study showed that the purity of levels hLLCs, indicated by SF-1/ZsGreen1 signals, were 75.16 ± 1.18% and 83.43 ± 1.66% at ID 22 and 33, respectively. This purity suggests that the overwhelming majority of signals in our experiments should come from hLLCs. Combining this model with a cell-binding assay and a SARS-CoV-2 spike pseudovector system [14], we successfully showed that SARS-CoV-2 spike pseudovectors could bind to and enter hLLCs. With further tracking of pseudovector-transduced hLLCs, we found that T biosynthesis in hLLCs was altered by SARS-CoV-2 spike binding and entry and could be partially rescued by SARS-CoV-2 inhibitors. Taking all the data together, we conclude that the SARS-CoV-2 virus could enter human Leydig cells and alter normal T biosynthesis.

### 4.1. T Production and SARS-CoV-2

We observed an acute increase in T production after SARS-CoV-2 spike pseudovector transduction in the hLLC model. This elevation is inconsistent with clinical reports that low T levels were found in the majority of hospitalized COVID-19 males [6,37]. The hiPSCs we used to generate hLLCs were donated by a young male without a hypogonadal history. Moreover, this hLLC model previously showed significant upregulation of T production in response to hCG stimulation [12]. It is likely that our observations here may be due to an acute response of hLLCs to SARS-CoV-2 entry, which can hardly be observed clinically and would be quickly dissipated by its negative feedback on the pituitary gland. In humans, T biosynthesis is regulated by the hypothalamic–pituitary–gonadal (HPG) axis [36]. Gonadotropin-releasing hormone (GnRH) stimulates luteinizing hormone (LH), which further stimulates the release of T from Leydig cells [36]. As lower levels of LH were also reported in COVID-19 males [6], it is possible that a reversible acute increase in T inhibited the release of either GnRH or LH. GnRH also triggers the secretion of follicle-stimulating hormone (FSH), which supports spermatogenesis [36]. The low levels of gonadotropins LH and FSH may be the result of Leydig cell dysfunction and spermatogenesis impairment [38].

Previous studies reported abnormal T or LH levels in COVID-19 patients versus those of healthy males [6], implying hypogonadal states are associated with COVID-19 infections. Moreover, T levels naturally decrease with advanced age in males, and lower levels are associated with various comorbidities, such as diabetes and other chronic health conditions [39]. Importantly, a bias towards older individuals was noted among COVID-19 patients who required hospitalization [39]. Consequently, a decline in T levels that is correlated with aging could exacerbate the outcomes of COVID-19. Thus, the lack of pre-COVID-19 comparative hormone data in these studies makes it challenging to study whether COVID-19 decreases T levels or whether existing hypogonadal states make males more vulnerable to COVID-19 [40]. Our data show an increased tendency for T production by hLLCs after SARS-CoV-2 spike pseudovector entry, indicating that pre-SARS-CoV2 blood samples of patients are necessary to confirm whether hypogonadism is a predisposing factor or a consequence of COVID-19. Higher immune system activation was found in COVID-19 patients and was associated with cholesterol reductions and the downregulation of T production by Leydig cells [37,41]. In real cases, the activation of the immune system and the decrease in cholesterol, which is the main source of T, may lead to impaired androgen synthesis [37]. However, in our culture system, the lack of immune cells and the consistent cholesterol source provided by fetal bovine serum make it impossible to observe inflammation-mediated cholesterol and T production reduction after SARS-CoV-2 entry. The inconsistency between the data indicates the shortcomings of employing single-cell-based experimental models to study COVID-19, especially in the human testis, which is composed of several interacting cell types that are mutually regulated. Therefore, co-cultures of various testicular cell types, such as macrophage/T cells with hLLCs, might be a more realistic model to study the pathological events in the COVID-19 testis.

It is also likely that the induction of testosterone production is due to pseudovirus entry and does not mimic actual SARS-CoV-2 infection. This is supported by the finding that spike entry does not reduce T immediately, but only after 3 days of pseudovirus transduction, suggesting that the effect on T production may be directly related to VSV transduction rather than the presence of SARS-CoV-2 proteins.

### 4.2. SARS-CoV-2 Entry into Leydig Cells

Targeting the entry of the virus by inhibiting SARS-CoV-2 receptors or proteases is an effective way to develop preventative vaccines [42]. Cell lines known to be highly susceptible to SARS-CoV-2, such as Vero E6 (also termed Vero 76), BGMK, and LLC-MK2, are commonly used to isolate or propagate SARS-CoV-2 and to screen antiviral drugs [43,44]. However, antiviral drugs against SARS-CoV-2 in these cell lines may not exert the same effects on other cell types or tissues, as different cell types showed diverse cellular tropism to SARS-CoV-2 [45]. To confirm the SARS-CoV-2 tropism of hLLCs, we tested the effects of ACE2 and TMPRSS2 inhibitors against spike pseudovector entry. These inhibitors showed differential effects on the SARS-CoV-2 entry between hLLCs and Vero E6 cells, suggesting tissue-specific intervention strategies should be considered against SARS-CoV-2 entry. Not surprisingly, the hLLC and H295-R entry pathways largely overlapped, as they are both steroid-producing cells and have many highly expressed genes in common [46]. Their steroidogenic activity may also contribute to their susceptibility to SARS-CoV-2. It is worth noting that males, who possess steroidogenic Leydig cells, are predisposed to be most severely affected by COVID-19. Moreover, aging men, whose T production is generally in decline, account for more deaths than either women or younger males [39,47]. In addition, the effects of SARS-CoV-2 infections on spermatogenesis raise concerns about infertility in men who have plans to conceive [48]. The demand for designing specific intervention strategies for targeting steroidogenic cells against COVID-19 is rising. The use of exogenous sex hormone and hormone receptor modulators for COVID-19 treatments might be a feasible way to diminish gender-based differences in outcomes for COVID-19 [49,50].

Cinanserin was found to target 3CL protease (3CL^pro^ or M^pro^) [26], the key protease of SARS-CoV-2 [51], which makes it an intriguing target for preventing SARS-CoV-2 infection. The 3CL protease primarily mediates coronavirus replication and transcription [52,53]. Our data showed that cinanserin significantly blocked SARS-CoV-2 spike pseudovectors, which are replication-deficient and do not include the SARS-CoV-2 genome. However, cinanserin could not significantly block the entry of VSVΔG vectors, implying that it mainly targets the spike protein. This result is consistent with the finding that cinanserin might target other SARS-CoV-2 components in addition to 3CL protease in blocking SARS-CoV-2 infections. Interestingly, cinanserin showed superiority in antiviral activity assays against SARS-CoV-2 compared to its performance in inhibiting 3CL protease activity [26]. VSVΔG-transduced hLLCs produced less T than bald vectors; however, the spike-pseudovector-transduced hLLCs produced more T than the bald vectors, ruling out the possibility that the elevation of T levels is due mainly to the presence of the VSVΔG genome. It is worth noting that our analysis of viral vector entry depends on the ratio of luminescent signals rather than absolute values. The capacity of cinanserin (3CLpro In) to block VSVΔG, as indicated by the luminescence intensity values, is higher than that of blocking spike pseudovectors, raising the possibility that cinanserin may target both VSVΔG and spike proteins. Moreover, the high concentration of VSVΔG vectors in hLLCs may have strong cytotoxicity, which would potentially counteract the stimulation of steroidogenesis. Nevertheless, our data also showed that cinanserin blocks SARS-CoV-2 entry more significantly than ACE2 and TMPRSS2 inhibitors in hLLCs and H295-R cells, suggesting cinanserin is a good candidate drug for preventing SARS-CoV-2 infections in steroidogenic cells.

Unlike the 99% antiviral effect of ACE2 inhibitor on Vero E6 cells, in hLLCs the ACE2 inhibitor was incapable of entirely blocking SARS-CoV-2 entry (blocking efficiency: 24.7 ± 8.2%), indicating that SARS-CoV-2 may enter hLLCs through other pathways. We, therefore, measured an additional six reported receptors or proteases of SARS-CoV-2. Among these proteins, NRP1, which can bind to furin-cleaved spike protein and potentiate the entry and infectivity of SARS-CoV-2 [28], is specifically expressed in hLLCs and human testes. Thus, EG00029, a known NRP1 inhibitor [54], as well as newly found small molecules that can interrupt the VEGF-A/NRP-1 pathway interfering with SARS-CoV-2 spike protein [55,56] will be intriguing candidates for us to explore more fully the entry mechanism of SARS-CoV-2 in Leydig cells and to develop antiviral drugs against SARS-CoV-2 infections in the testis. Moreover, CTSB and CTSL are also potential targets for preventing infections of the SARS-CoV-2 omicron variant, as increased CTSB/CTSL levels and decreased use of TMPRSS2 were found in omicron variant infections [57]. Consistently, our data showed low expression of TMPRSS2 in hLLCs, low efficacy of TMPRSS2 inhibitor blocking SARS-CoV-2 spike entry, and high expression of CTSB/CTSL in steroidogenic cells, suggesting that the endocrine system may be more vulnerable to SARS-CoV-2 omicron infections. E64d, a CTSB inhibitor [58], and MDL28170, a CTSL inhibitor, both showed therapeutic antiviral effects targeting SARS-CoV-2 entry [59] and are excellent candidates against SARS-CoV-2 omicron infections.

### 4.3. Future Work on SARS-CoV-2 Infection and the Testis

The SARS-CoV-2 spike pseudovirus entry assay enabled us to investigate hLLC tropism and entry mechanisms of SARS-CoV-2, but it was incapable of mimicking the whole process of SARS-CoV-2 infection. It is possible that SARS-CoV-2 replication and propagation can further impair functions or cause apoptosis, resulting in a T level decline, which our current studies cannot reveal. In the future, the employment of the live SARS-CoV-2 virus, including various variants to track their effects on hLLCs, is required. In addition, the transduction of pseudovectors on cultured cells in vitro is an artificial system that may not be able to recapitulate the physiological infective dose of an authentic virus in vivo. The doses of the pseudovectors for transduction on VeroE6 and hLLCs in this study were selected to achieve comparable transduction levels to the reported data from a broad panel of SARS-CoV-2 spike pseudovector-susceptible cell lines (~10^4^ to 10^6^ RLU) in our or others’ previous publications [14,60,61] and were not based on physiologically measured infective titers. Considering that a single cell type cannot reflect the response of the whole endocrine system to viral attack, performing live SARS-CoV-2 assays in an animal model (mouse or hamster) to investigate the effect of SARS-CoV-2 infection on the testis is needed. Moreover, as we only tracked the T biosynthesis of hLLCs for a limited time, the development a long-term hLLC model to follow the overall host response and pathological changes after infection is also needed. In addition, as there are prolonged complications from SARS-CoV-2 infection, such as shortness of breath, depression, and chronic memory loss, known as “long COVID” [62,63], the long-term tracking of infected hLLCs or animal models will be key to cracking the mystery of long COVID [64] and should enable us to find answers for some of the questions surrounding the long-term effects.

## 5. Conclusions

Our study provides direct evidence of SARS-CoV-2 entry into a human Leydig cell model and new insights into the high percentage of hypogonadal males within the COVID-19 patient population. Combining a SARS-CoV-2 spike pseudovector system and inhibitor screening assays results in a better understanding for exploring Leydig cell tropism of SARS-CoV-2 and SARS-CoV-2 entry mechanisms and pathways, and more importantly is a robust tool for antiviral drug screening for the human testis.

## Figures and Tables

**Table 1:** The qPCR primers (IDT).

Gene	Primer FW	Primer RW
ACE2	ACAGTCCACACTTGCCCAAAT	TGAGAGCACTGAAGACCCATT
TMPRSS2	GTCCCCACTGTCTACGAGGT	CAGACGACGGGGTTGGAAG
TMPRSS4	GACAAACAGCACGTCTGTGGA	GCCTGAGAAAGTGAGTGGGAA
NRP1	ACGTGGAAGTCTTCGATGGAG	CACCATGTGTTTCGTAGTCAGA
Basigin	GAAGTCGTCAGAACACATCAACG	TTCCGGCGCTTCTCGTAGA
DPP4	GGGTCACATGGTCACCAGTG	TCTGTGTCGTTAAATTGGGCATA
CTSB	AGAGTTATGTTTACCGAGGACCT	GATGCAGATCCGGTCAGAGA
CTSL	AGGGTCAGTGTGGTTCTTGTTG	TGAGATAAGCCTCCCAGTTTTC
Furin	CCTGGTTGCTATGGGTGGTAG	AAGTGGTAATAGTCCCCGAAGA
S18	ATTAAGGGTGTGGGCCGAAG	TGGCTAGGACCTGGCTGTAT

**Table 2:** The antibodies used in this study.

Antibody	Host	Company	Catalogue No.	Dilution	Application
ACE2	Rabbit	Abcam	ab108252	1:1000	WB
TMPRSS2	Rabbit	Abcam	ab109131	1:1000	WB
TMPRSS4	Rabbit	ThermoFisher Scientific	11283-1-AP	1:500	WB
NRP1	Rabbit	Abcam	ab81321	1:1000	WB
Basigin	Rabbit	Cell Signaling	13287S	1:1000	WB
DPP4	Mouse	ThermoFisher Scientific	TA500733	1:500	WB
CTSB	Rabbit	Cell Signaling	31718T	1:1000	WB
CTSL	Mouse	ThermoFisher Scientific	BMS1032	1:1000	WB
Furin	Mouse	R&D	MAB15032-SP	1:500	WB
GAPDH	Rabbit	Cell Signaling	2118L	1:1000	WB
SARS-CoV-2 Nucleocapsid	Rabbit	ThermoFisher Scientific	MA5-29982	1:50	IFC
Prolactin R	Mouse	Novus Bio	NB300-561	1:200	IFC
Goat Anti-Mouse IgG	Goat	LI-COR Biosciences	926-80010	1:4000	WB
Goat Anti-Rabbit IgG	Goat	LI-COR Biosciences	926-80011	1:4000	WB
Goat Anti-Rabbit IgG	Goat	ThermoFisher Scientific	A21244	1:300	IFC

**Table 3:** Inhibitors potentially targeting receptors or proteases of SARS-CoV-2.

Name	Functions	Concentrations	Company	Catalogue No.
MLN-4760	ACE2 inhibitor	37 nM (16 ng/mL)	Millipore Sigma	530616001
Camostat mesylate	TMPRSS2 inhibitor	50 µM (24 µg/mL)	Millipore Sigma	SML0057-10MG
Cinanserin hydrochloride	3CL proteases inhibitor	40 µM (15 µg/mL)	Tocris	0460

## Data Availability

The data that support the findings of this study are available in the Methods and Results sections of this article.

## Supplementary Materials

The following supporting information can be downloaded at: https://www.mdpi.com/article/10.3390/cells12081198/s1. Figure S1: Fluorescent immunocytochemistry of prolactin receptor and second antibodies in the hamster testis. Figure S2: Protein expression profiles of different cell types and human testes lysates. Figure S3: Morphology of hLLCs and ZsGreen1 signals in hLLCs. Figure S4: FACS results of hLLCs at different induction time points.
