# Supplementary material for: SARS-CoV-2 Enters Human Leydig Cells and Affects Testosterone Production In Vitro
Source: Cells. 2023 Apr 20;12(8):1198. doi: 10.3390/cells12081198 (PMC10136776; doi:10.3390/cells12081198)
Supplement: Supplementary file 1 [file cells-12-01198-s001.zip › cells-2286526-supplementary.pdf]

Supplemental Material

SARS-CoV-2 Enters Human Leydig Cells and Affects Testosterone Production in Vitro

Lu Li[1], Chantal M. Sottas[1], Hsu-Yu Chen[2], Yuchang Li[1], Haoyi Cui[1], Jason S. Villano[3], Joseph L. Mankowski[3], Paula M. Cannon[2] and Vassilios Papadopoulos[1]

[1]Department of Pharmacology and Pharmaceutical Sciences, Alfred E. Mann School of Pharmacy and Pharmaceutical Sciences, University of Southern California, Los Angeles, CA 90089;

[2]Department of Molecular Microbiology and Immunology, Keck School of Medicine, University of Southern California, Los Angeles, CA 90033;

[3]Departments of Molecular and Comparative Pathobiology, Pathology and Neurology, The Johns Hopkins School of Medicine, Baltimore, MD 21205

Four Figures (S1-S4)

Fig. S1. Fluorescent immunocytochemistry of prolactin receptor and 2nd antibodies in the hamster testis.

(A) Prolactin receptor (prolactin R) antibodies can detect Leydig cells (green signals) in the hamster testis. (B) Non-specific signals of 2nd antibodies (green and red signals) overlapped with nuclear, indicated by DAPI signals. The non-specific signals were extremely bright.

Fig. S2. Protein expression profiles of different cell types and human testes lysates.

(A) Western immunoblot analyses of ACE2, TMPRSS2, and GAPDH expression in hiPSC, hLLC, H295-R, and Vero E6 cells. Human testicular lysates were used as a positive control. 125 kDa, 50 kDa, and 37 kDa bands were detected by ACE2, TMPRSS2, and GAPDH antibodies in different cell types. Red squares indicate the target bands. (B-C) Quantitative analyses of ACE2 and TMPRSS2 expression in hiPSC, hLLC, and H295-R cells when normalized to GPADH. Data are presented as mean ± SD, n = 3. Not significant (n.s.) $P > 0.05$; * $P < 0.05$; ** $P < 0.01$.

Fig. S3. Morphology of hLLCs and ZsGreen1 signals in hLLCs.

The detailed enlarged phase contrast and fluorescent microscopy images of hLLCs. (A) Green fluorescent signals indicate ZsGreen1-tagged SF-1 expression. The red, blue, and green arrows point to hLLCs with intense, low/scattered, and negligible ZsGreen 1 signals, respectively. (B) After FACS, hLLCs with different ZsGreen1 intensities were cultured under hLLC induction conditions and observed under a fluorescent microscope.

Fig. S4. FACS results of hLLCs at different induction time points.

FACS analyses classified mixed hLLCs into a high-intensity group (HI), low-intensity group (LI), and no signal group (NS). Early mesenchymal progenitors (EMPs) cultured in hLLC induction medium without DOX were used as negative controls (NC). Three replicates of NC and hLLCs collected at ID22, 32, and 82 were used for FACS analyses. The results of one representative biological replicate for each group are shown in Figure 5B. The other two replicates are shown here.

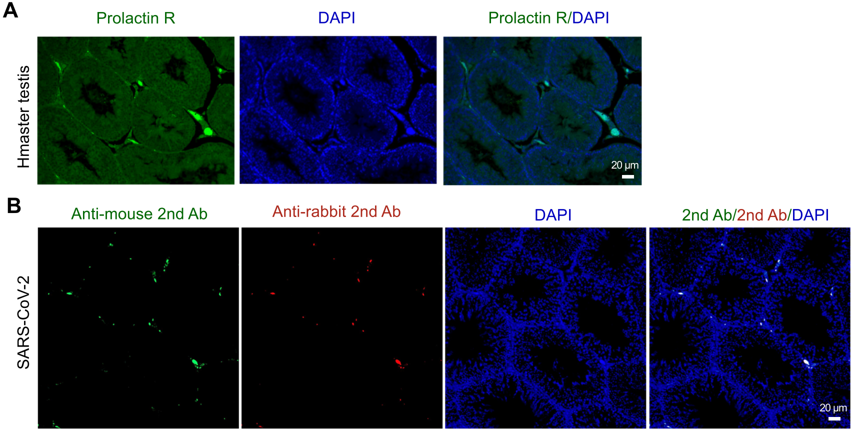
A
Prolactin R
DAPI
Prolactin R/DAPI
Hmaster testis
20 µm
B
Anti-mouse 2nd Ab
Anti-rabbit 2nd Ab
DAPI
2nd Ab/2nd Ab/DAPI
SARS-CoV-2
20 µm

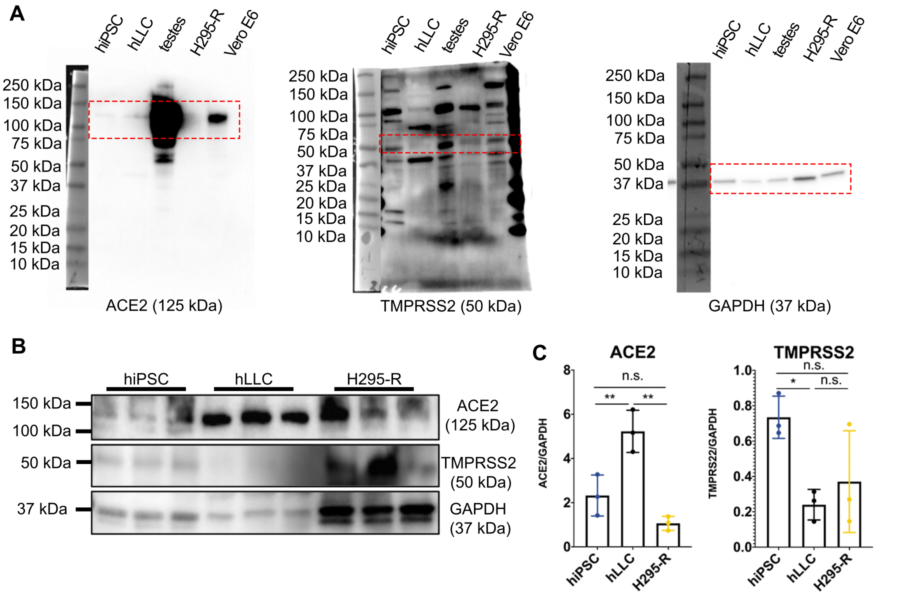

A
hiPSC
hLLC
testes
H295-R
Vero E6
250 kDa
150 kDa
100 kDa
75 kDa
50 kDa
37 kDa
25 kDa
20 kDa
15 kDa
10 kDa
ACE2 (125 kDa)
TMPRSS2 (50 kDa)
GAPDH (37 kDa)
B
hiPSC
hLLC
H295-R
150 kDa
100 kDa
50 kDa
37 kDa
ACE2 (125 kDa)
TMPRSS2 (50 kDa)
GAPDH (37 kDa)
C
ACE2
ACE2/GAPDH
n.s.
**
**
TMPRSS2
TMPRS22/GAPDH
n.s.
*
n.s.
hiPSC
hLLC
H295-R

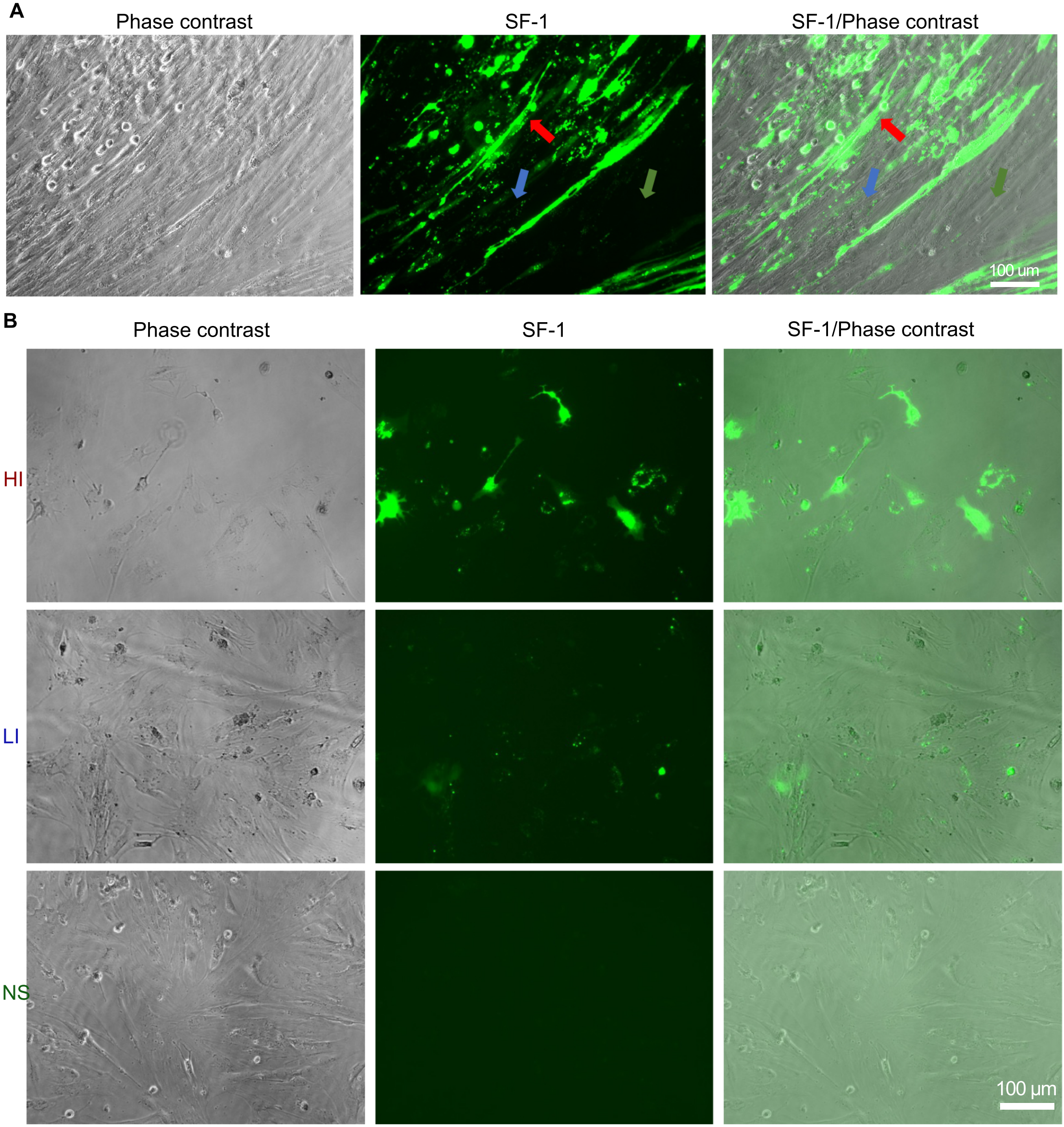
A
Phase contrast
SF-1
SF-1/Phase contrast
100 um
B
Phase contrast
SF-1
SF-1/Phase contrast
HI
LI
NS
100 μm

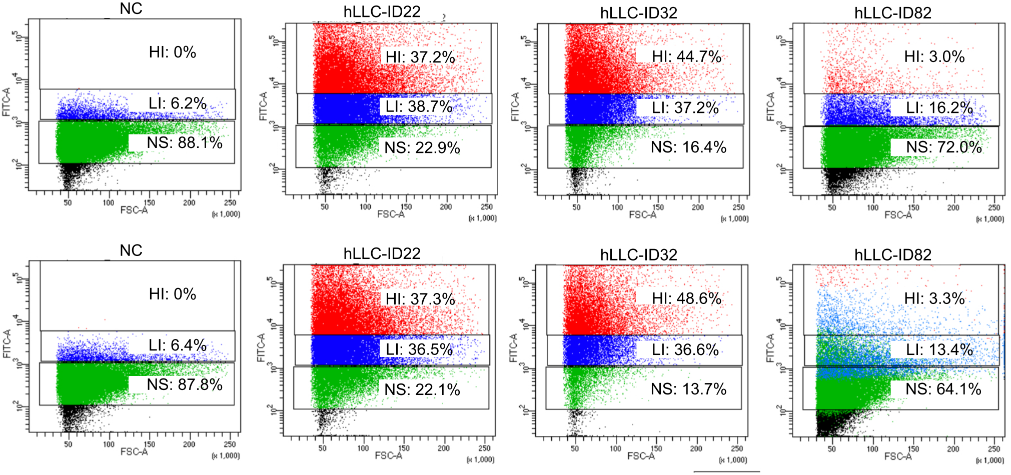
NC
HI: 0%
LI: 6.2%
NS: 88.1%
hLLC-ID22
HI: 37.2%
LI: 38.7%
NS: 22.9%
hLLC-ID32
HI: 44.7%
LI: 37.2%
NS: 16.4%
hLLC-ID82
HI: 3.0%
LI: 16.2%
NS: 72.0%
NC
HI: 0%
LI: 6.4%
NS: 87.8%
hLLC-ID22
HI: 37.3%
LI: 36.5%
NS: 22.1%
hLLC-ID32
HI: 48.6%
LI: 36.6%
NS: 13.7%
hLLC-ID82
HI: 3.3%
LI: 13.4%
NS: 64.1%
FITC-A
FSC-A
(x 1,000)
